# COHORT DIFFERENCES AND 4-YEAR CHANGE IN EVERYDAY DISCRIMINATION AMONG OLDER AMERICANS

**DOI:** 10.1093/geroni/igz038.1169

**Published:** 2019-11-08

**Authors:** Felicia V Wheaton

**Affiliations:** Bethune-Cookman University, Daytona Beach, Florida, United States

## Abstract

Experiences of everyday discrimination, such as being treated with less courtesy or respect, are associated with poorer mental and physical health in later life. Yet not much is known about cohort differences in experiences of everyday discrimination, nor how subjective reports of discrimination change over time among older adults. This study assessed cohort differences and 4-year change in everyday discrimination using data from the 2006 and 2010 waves of the Health and Retirement Study (HRS), a nationally-representative study of Americans 50+. In both waves, participants were asked about 5 possible experiences of everyday discrimination: for example, “In your day-to-day life, how often have any of the following things happened to you? You receive poorer service than other people at restaurants and stores” and could respond from 0=never to 5=almost every day. Average everyday discrimination was lower for older cohorts compared with younger cohorts in both 2006 and 2010. Paired-samples t-tests showed that average everyday discrimination declined significantly over the 4-year period. However, it is unclear if changes are due to period or age effects. Implications of these findings will be discussed.

